# 5-Methoxyquinoline Derivatives as a New Class of EZH2 Inhibitors

**DOI:** 10.3390/molecules20057620

**Published:** 2015-04-27

**Authors:** Pu Xiang, Hui Jie, Yang Zhou, Bo Yang, Hui-Juan Wang, Jing Hu, Jian Hu, Sheng-Yong Yang, Ying-Lan Zhao

**Affiliations:** 1 State Key Laboratory of Biotherapy and Cancer Center, West China Hospital, Sichuan University, and Collaborative Innovation Center for Biotherapy, Chengdu 610041, China; 2 West China School of Pharmacy, Sichuan University, Chengdu 610041, China; 3 The Second Division of Hepatobiliary Surgery, Center of PLA, Center of General Surgery of PLA, General Hospital of Chengdu Military Region, Chengdu 610083, China

**Keywords:** histone methyltransferase, Enhancer of Zeste Homologue 2 (EZH2), quinolines, anticancer activity

## Abstract

A series of quinoline derivatives was synthesized and biologically evaluated as Enhancer of Zeste Homologue 2 (EZH2) inhibitors. Structure-activity relationship (SAR) studies led to the discovery of 5-methoxy-2-(4-methyl-1,4-diazepan-1-yl)-*N*-(1-methylpiperidin-4-yl)quinolin-4-amine (**5k**), which displayed an IC_50_ value of 1.2 μM against EZH2, decreased global H3K27me3 level in cells and also showed good anti-viability activities against two tumor cell lines. Due to the low molecular weight and the fact that no quinoline derivative has been reported as an EZH2 inhibitor, this compound could serve as a lead compound for further optimization.

## 1. Introduction

Enhancer of Zeste Homologue 2 (EZH2) is a member of the histone-lysine *N*-methyltransferase (HKMT) family, which methylates K9 and K27 of histone H3, leading to transcriptional repression of the affected target genes. EZH2 has been found to be over-activated or over-expressed in many cancer types, such as lymphoma, colon, prostate, breast, and lung cancer [1,2]. The over-activation or over-expression of EZH2 has been linked to aberrant repression of some tumor suppressor genes, and is often implicated in tumor progression and correlates with poor prognosis [3,4]. Therefore, EZH2 has been highlighted as a promising intervention target for cancer therapy.

**Figure 1:**
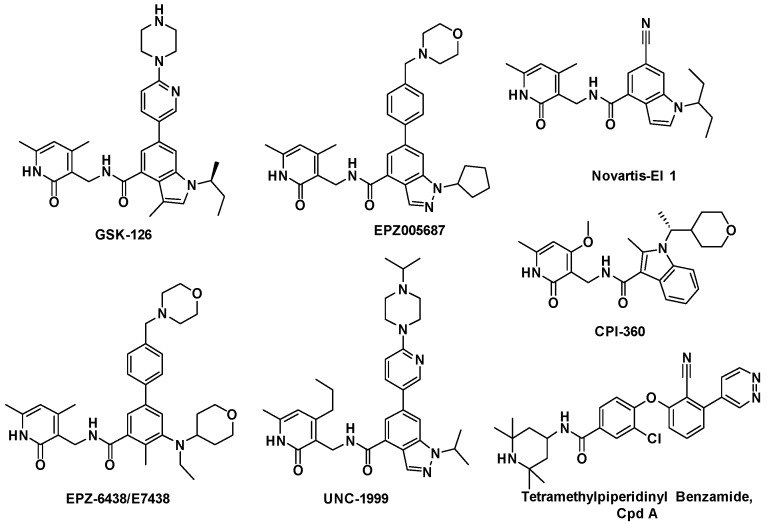
Typical EZH2 inhibitors currently reported.

**Figure 2:**
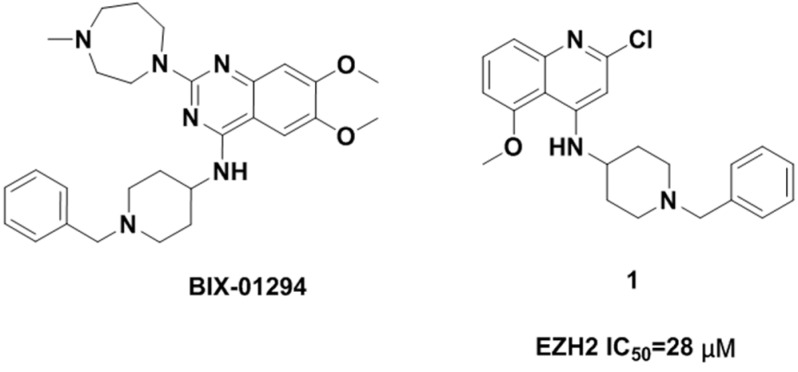
Structure of BIX-01294 and hit compound **1**.

Due to the potential therapeutic value of EZH2 inhibitors in the treatment of cancer, many pharmaceutical companies and academic institutes have recently been involved in the development of small molecule inhibitors of EZH2. To date, a number of EZH2 inhibitors (see Figure 1) have been reported [5,6]. Unfortunately, the known EZH2 inhibitors have very limited scaffold structures. We thus decided to discover new classes of EZH2 inhibitors with scaffolds different from those of known EZH2 inhibitors. BIX-01294 (2-(hexahydro-4-methyl-1*H*-1,4-diazepin-1-yl)-6,7-dimethoxy-*N*-[1-(phenylmethyl)-4-piperidinyl]-4-quinazolinamine, Figure 2) is a well-known histone methyltransferase G9a/GLP inhibitor containing a 1-benzylpiperidin-4-ylamino group [7]. Simultaneously, quinoline derivatives are useful in diverse applications including pharmaceuticals and are available as drugs today, and have never been reported as EZH2 inhibitors. Thus we considered following the example of BIX-01294 to develop novel EZH2 inhibitors. We synthesized compound **1** (*N*-(1-benzylpiperidin-4-yl)-2-chloro-5-methoxyquinolin-4-amine, Figure 2) and then verified its activity against EZH2. This compound had a half maximal inhibitory concentration (IC_50_) value of 28 μM against EZH2. Obviously the potency of this compound was too low and needed further improvement. Therefore in this investigation we carried out structural optimization to compound **1**. A series of quinoline derivatives were synthesized, and the structure–activity relationships (SAR) of these compounds will also be discussed.

## 2. Results and Discussion

### 2.1. Chemistry

**Scheme 1:**
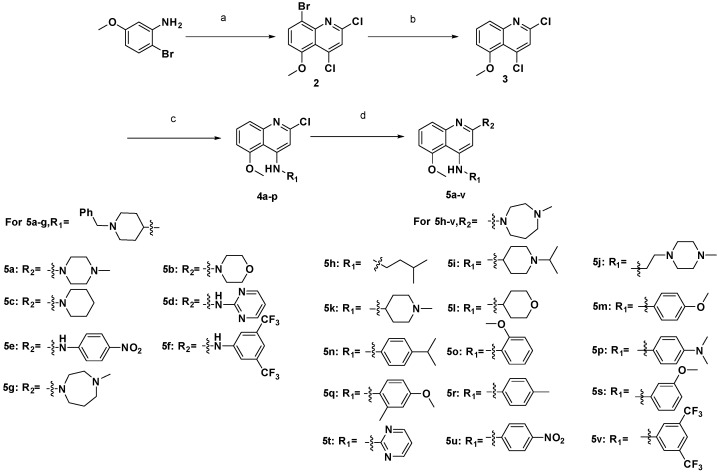
Synthetic routes of compounds **5a**–**v**.

The general synthetic routes employed for the preparation of 5-methoxyquinoline derivatives **5a**–**v** are outlined in Scheme 1. The intermediate 8-bromo-2,4-dichloro-5-methoxyquinoline (**2**) was prepared by cyclization of 2-bromo-5-methoxyaniline and malonic acid, using POCl_3_ as catalyst and solvent [8]. Then the bromine in the 8-positionof **2** was removed by *n*-BuLi in THF and CH_3_OH to give compound **3** [9], which reacted with a variety of primary or secondary amines at the 4-position of the quinoline moiety to produce compounds **4a**–**p** by a nucleophilic substitution reaction [10]. Finally, the final products **5a**–**v** were obtained by another nucleophilic substitution reaction of **4a**–**p** with amines at the 2-position of the quinoline moiety in *i*-PrOH under microwave conditions, using TFA as an acid catalyst [11].

*Reagents and conditions*: (**a**) malonic acid, phosphorus oxychloride, reflux, 120 °C, 16 h; (**b**) n-Butyllithium, tetrahydrofuran, methanol, −78–0 °C, 2 h; (**c**) 1-Methyl-2-pyrrolidinone, ethyldiisopropylamine, 150 °C, R_1_NH_2_, 4 h; (**d**) trifluoroacetic acid, isopropanol, 160 °C, R_2_H, microwaves, 30 min.

**Scheme 2:**
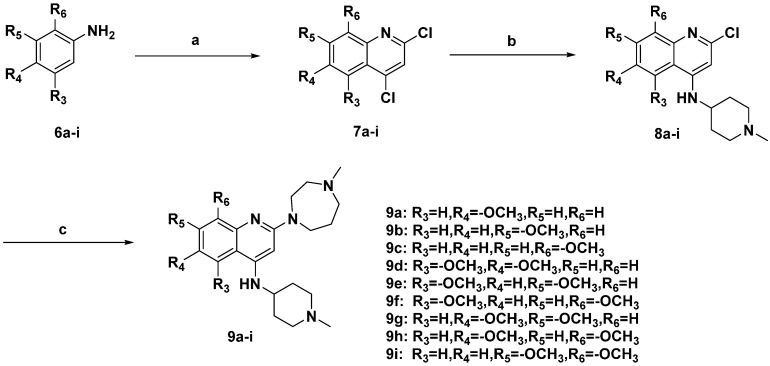
Synthetic routes of compounds **9a**–**i**.

The general routes for the preparation of quinoline derivatives **9a**–**i** are outlined in Scheme 2. Phenylamines **6a**–**i** with single or double substituted methoxy groups were cyclized with malonic acid, using POCl_3_ as catalyst and solvent, to give intermediates **7a**–**i**, which reacted with 1-methylpiperidin-4-amine at the 4-position of the quinoline moiety to produce compounds **8a**–**i** by a nucleophilic substitution reaction. Finally, the products **9a**–**i** were obtained by another nucleophilic substitution reaction of **8a**–**i** with 1-methyl-1,4-diazepane at the 2-position of the quinoline moiety in *i*-PrOH under microwave conditions, using TFA as an acid catalyst.

*Reagents and conditions*: (**a**) malonic acid, phosphorus oxychloride, reflux, 120 °C, 16 h; (**b**) 1-methyl-2-pyrrolidinone, ethyldiisopropylamine, 150 °C, 1-methylpiperidin-4-amine, 4 h; (**c**) trifluoroacetic acid, isopropanol, 160 °C, 1-methyl-1,4-diazepane, microwaves, 30 min.

### 2.2. SAR Analyses

The SAR analyses of the quinoline derivatives were based on both enzymatic and cellular assays, in which the enzymatic activities were determined by the AlphaLISA method, and the cell growth inhibition potencies were evaluated on the HCT15 and MD-MB-231 cell lines by the MTT method.

The SAR analysis started by varying substituents of the 2-position of quinoline moiety with the 4-position being substituted by 1-benzylpiperidin-4-amine. We first synthesized eight compounds (**1**, **5a**–**g**) with each one bearing different substituents or no substituent at the 2-position of the quinoline. Bioactivities of these compounds are summarized in Table 1. Among these compounds, **5g**, which contains a 1-methyl-1,4-diazepane group at the 2-position, was the most active compound at both the enzymatic and cellular levels. Though compound **5c**, which bears a piperidine group at the 2-position, had a good activity at the cellular level, it did not show notable activity at the enzymatic level (IC_50_ > 10 μM). Compounds **5d**–**f**, which contain a primary amine at the 2-position of quinoline moiety, only exhibited relatively weak potency.

**Table 1:** Bioactivities of compounds **1**, **5a**–**g**.

Compound	IC_50_^a^ (μM)		
	EZH2(AlphaLISA)	HCT15	MDA-MB-231
**GSK-126**	0.001	N.T.	N.T.
**1**	28.0 ± 2.23	26.0 ± 2.68	>20.0
**5a**	16.4 ± 1.24	12.3 ± 0.95	11.9 ± 1.83
**5b**	23.0 ± 2.06	15.6 ± 1.27	14.2 ± 1.97
**5c**	32.0 ± 2.78	7.80 ± 0.46	5.60 ± 0.14
**5d**	17.5 ± 0.96	>20	>20
**5e**	29.4 ± 1.01	17.8 ± 1.27	14.9 ± 2.20
**5f**	21.6 ± 2.35	10.2 ± 1.24	>20
**5g**	6.6 ± 0.49	3.78 ± 0.07	4.56 ± 0.24

^a^ Values are means of at least three measurements. N.T.: Not Tested.

We then fixed the 2-position of the quinoline nucleus with a 1-methyl-1,4-diazepane unit as the optimal group, and varied the 4-position of the quinoline moiety. A total of 15 compounds were synthesized. Bioactivities of these compounds are listed in Table 2. From Table 2, we can see that **5k**, which contains a 1-methylpiperidin-4-amine group at the 4-position, was the most active compound in the enzymatic assay, with an IC_50_ value of 1.2 μM. It also showed a good cellular potency against HCT15 and MDA-MB-231, with IC_50_ values of 5.6 μM and 2.45 μM, respectively. Obviously, the bioactivities of **5k** at both the enzymatic and cellular levels were increased by more than 20-fold compared with those of **1**.

**Table 2:** Bioactivities of compounds **5h**–**v**.

Compound	IC_50_^a^ (μM)		
	EZH2(AlphaLISA)	HCT15	MDA-MB-231
**5h**	11.5 ± 1.21	5.46 ± 0.85	4.31 ± 0.57
**5i**	17.4 ± 1.07	>20	15.7 ± 1.27
**5j**	4.20 ± 0.20	18.6 ± 2.20	12.4 ± 0.95
**5k**	1.20 ± 0.11	7.60 ± 0.46	2.45 ± 0.24
**5l**	2.80 ± 0.21	15.5 ± 0.20	2.40 ± 0.49
**5m**	19.7 ± 2.56	18.9 ± 1.97	>20
**5n**	26.4 ± 2.04	14.6 ± 1.27	13.2 ± 2.53
**5o**	17.5 ± 1.13	>20	>20
**5p**	27.6 ± 0.97	17.4 ± 1.54	16.2 ± 1.67
**5q**	34.8 ± 1.54	16.9 ± 0.36	14.5 ± 1.23
**5r**	39.7 ± 1.26	12.9 ± 0.89	>20
**5s**	16.8 ± 0.94	19.6 ± 0.56	16.9 ± 1.06
**5t**	35.3 ± 1.61	>20	>20
**5u**	26.2 ± 2.54	17.8 ± 1.27	14.9 ± 2.20
**5v**	34.1 ± 2.22	10.2 ± 1.24	>20

^a^ Values are means of at least three measurements.

We further explored the possible effect of a methoxy group at different substitution positions of the quinoline moiety. Single or double methoxy groups were introduced to the 5, 6, 7 or 8-positions of the quinoline moiety, which led to compounds **9a**–**i**. As shown in Table 3, compound **9a** (6-methoxy substituted), **9d** (5,6-dimethoxy substituted), **9e** (5,7-dimethoxy substituted), and **9f** (5,8-dimethoxy substituted) displayed a slightly decreased potency in both enzymatic and cellular assays compared with **5k**, while the other compounds exhibited a greatly decreased activity. This indicated that methoxy groups at the 5-position might be more important for the potency of the compounds, and single or double substitutions at other positions would not contribute to the potency.

**Table 3:** Bioactivities of compounds **9a**–**i**.

Compound	IC_50_^a^ (μM)		
	EZH2(AlphaLISA)	HCT15	MDA-MB-231
**9a**	8.40 ± 0.34	10.9 ± 0.35	14.6 ± 2.24
**9b**	18.4 ± 1.65	>20	>20
**9c**	20.1 ± 0.85	>20	>20
**9d**	5.60 ± 0.45	8.60 ± 2.20	12.4 ± 0.95
**9e**	6.70 ± 0.36	10.6 ± 0.83	12.5 ± 0.62
**9f**	9.20 ± 0.51	15.5 ± 0.53	12.4 ± 0.49
**9g**	17.8 ± 2.07	15.9 ± 1.85	>20
**9h**	14.9 ± 1.71	19.8 ± 1.27	13.2 ± 1.64
**9i**	13.7 ± 1.32	>20	>20

^a^ Values are means of at least three measurements.

### 2.3. Selectivity of **5k**

To examine the selectivity of **5k**, we measured the activities of **5k** against several selected protein methyltransferases, including SMYD3, G9a, SUV39H1, SETDB1, PRMT1 and SETD8. As shown in Table 4, compound **5k** showed very weak activity against the selected protein methyltransferases, indicating a good selectivity for EZH2.

**Table 4:** Inhibition activity of **5k** against EZH2 and several other selected histone methyltransferases.

Targets	EZH2	SMYD3	G9a	SUV39H1	SETDB1	PRMT1	SETD8
IC_50_^a^ (μM)	1.20 ± 0.11	35.4 ± 1.97	82.6 ± 2.23	21.2 ± 0.28	27.0 ± 1.36	28.8 ± 1.25	40.0 ± 2.71

^a^ Values are means of at least three measurements.

### 2.4. Analysis of H3K27 Methylation in MDA-MB-231 Cell Line Treated with **5k**

**Figure 3:**
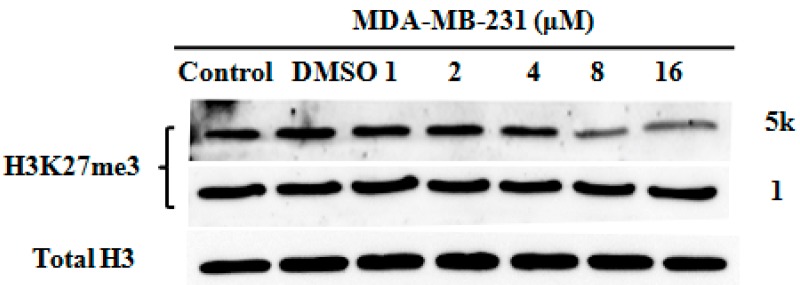
Analysis of H3K27 methylation in MDA-MB-231 cell line treated with **1** and **5k**. Evaluation of H3K27me3 following treatment for 72 h. Total H3 is shown as a loading control.

The main catalytic activity of EZH2 is methylating histone 3 lysine 27 (H3K27), so evaluation of H3K27me3 was conducted through a western blotting assay. As shown in Figure 3, the H3K27me3 level of MDA-MB-231cell treated with compound **5k** was obviously down-regulated. These results indicated that compound **5k** inhibited the methyltransferase activity of EZH2.

### 2.5. The Possible Binding Mode of **5k** with EZH2

**Figure 4:**
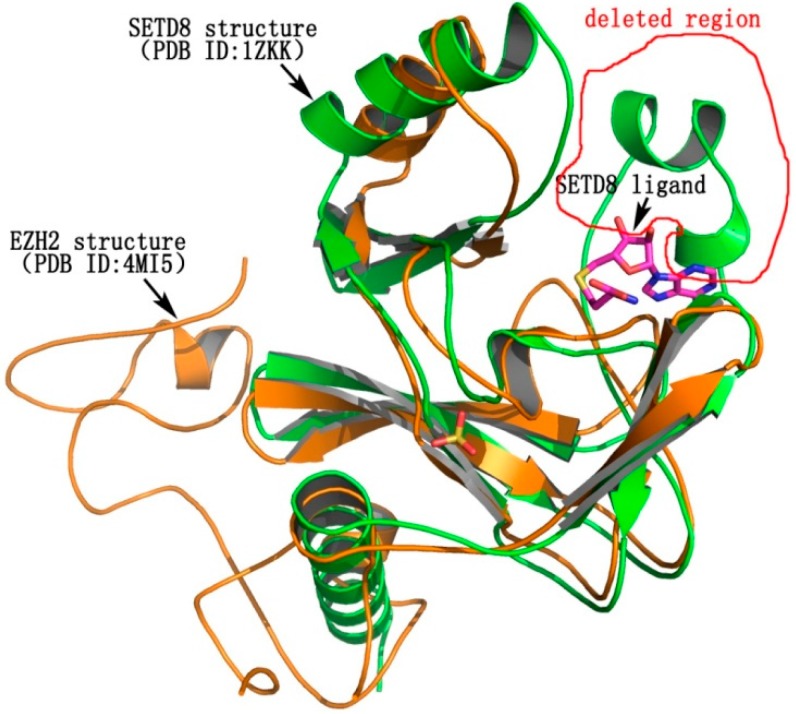
The homology of EZH2 (PDB ID: 4MI5) and SETD8 (PDB ID: 1ZKK). EZH2 is shown in yellow, while SETD8 is shown in green. The deleted region in the active binding pocket of EZH2 is circled in red.

**Figure 5:**
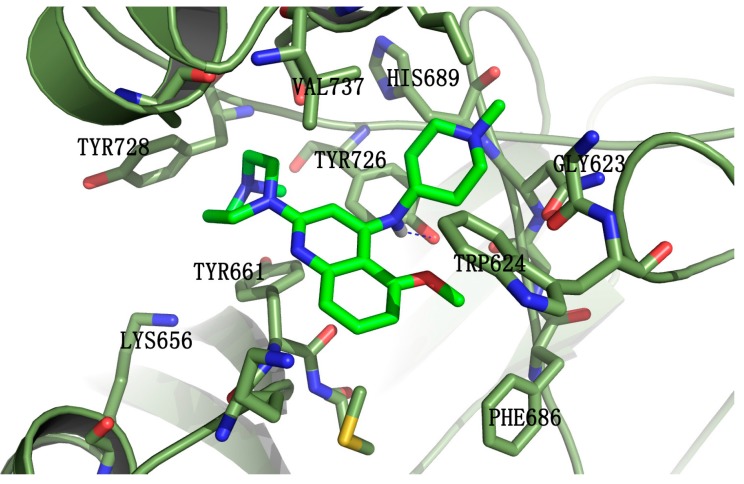
Predicted binding mode of compound **5k** with the SET-domain of EZH2. Compound **5k** is shown in green. EZH2 is represented by ribbons with the interacting residues represented as sticks. Hydrogen-bonding is drawn as dashed blue.

Molecular docking was employed to understand the binding mode of the most potent compound, **5k**, with EZH2. Some of the amino acid residues in the active binding pocket of EZH2 are deleted in the crystal structure (PDB ID: 4MI5). Hence this crystal structure is not an ideal receptor structure, and it could not be used in molecular docking studies. Therefore a complete structure containing all the amino acid residues in the active binding pocket should be constructed by the homology modeling method. The homology model of the EZH2 active pocket was built with the SETD8 X-ray crystal structure (PDB ID: 1ZKK) because of their homology (Figure 4). Figure 5 shows a predicted binding mode of **5k** with the SET domain of EZH2. Compound **5k** occupies the substrate binding groove, and the hydrogen of the secondary amino group at the 4-position of the quinoline ring indeed forms a hydrogen bond with TYR726.

## 3. Experimental Section

### 3.1. General

The human cancer cell lines HCT15 and MDA-MB-231 were purchased from the American Type Culture Collection (ATCC, Rockville, MD, USA). Dulbecco’s modified Eagle medium (DMEM) and RPMI 1640 were purchased from Gibco (Grand Island, NY, USA). Fetal bovine serum (FBS) was obtained from Hyclone (Logan, UT, USA). The purity of compound screened in biological assays was determined to be >98% by HPLC analysis. A Symmetry C_18_ (75 mm × 4.6 mm, i.d. 3.5 μm) (Waters, Milford, MA, USA) was used with acetonitrile and HPLC-grade water as mobile phase. ^1^H-NMR were recorded at 400 MHz, mass spectra (MS) were measured by a Q-TOF Premier (Micromass, Manchester, UK) mass spectrometer utilizing electrospray ionization (ESI).

### 3.2. AlphaLISA Technology Assay for EZH2

The AlphaLISA assay and the enzyme selectivity assay were performed by Shanghai ChemPartner Co., Ltd. (Shanghai, China). Briefly, compounds were transferred to the assay plate (OptiPlate-384, PerkinElmer, Waltham, MA, USA) with 3-fold dilution by Echo (compound total volume 100 nL), and 5 μL enzyme solution (final concentration was 2 nM) or pH 9.0 TRIS-based assay buffer (for Min well) to the assay plate were added, and centrifuged at 1000 rpm for 1 min. The assay plate was incubated for 15 min at RT prior to initiation of the reaction by addition of 5 μL biotinylated peptide/SAM mix (final concentrations were 100 nM and 3000 nM, respectively). The plate was covered with TopSeal-A film and incubated for 1 h at RT after centrifuging at 1000 rpm for 1 min (DMSO final concentration 1%). Acceptor beads (15 μL) and donor beads mixed were added to stop the enzymatic reaction. After incubating for 1 h again in subdued light, the assay plate was read in Alpha mode on an EnVision instrument (PerkinElmer, Waltham, MA, USA). GSK-126 was used as the positive control.

### 3.3. MTT Assay

Briefly, cells were seeded in 96-well plates and cultured for 24 h, followed by treatment with the compounds for 96 h. Ten microliters of 10 mg/mL MTT was added per well and incubated for another 2.5 h at 37 °C. Then the supernatant fluid was removed and 150 μL/well DMSO was added for 15–20 min. The absorbance (OD) of each well was measured at 570 nm, using a SpectraMAX M5 microplate spectrophotometer (Molecular Devices, CA, USA).

### 3.4. Western Blotting Assay of H3K27me3 Status

The western blotting assay of H3K27 status was performed as previously described [12].

### 3.5. Homology Modeling, Molecular Docking and Molecular Dynamic (MD) Simulation

The homology model of the SET domain of EZH2 was built using the *Build Homology Model* module of Discovery Studio (Accelrys, San Diego, CA, USA). For this step, the MODELER program was applied. The SET domain of N-lysine methyltransferase SETD8 (PDB accession code1ZKK) was used as template, because of its homology to EZH2, and similar structure in the SET domain.

All the molecular docking simulation is performed using the GOLD program in Discovery Studio. GOLD is a genetic algorithm-based molecular docking method that uses the CHARMM force field. All the molecules were prepared before docking using the prepare ligands protocol in Discovery Studio. The docking parameters are the default setting.

All the molecular dynamics (MD) simulations of the protein-ligand complex obtained by docking was carried out using the Standard Dynamics Cascade protocol in the Discovery Studio environment. For the Standard Dynamics Cascade, the CHARMM force field was employed for the protein and the GAFF force field for small molecules. During the simulation, five stages was experienced, which according to the order, are the minimization 1 using the robust steepest descent algorithm, minimization 2 using the conjugate gradient method, 10 ps heating stage (the temperature was changed from 50 to 300 K), 10 ps Equilibration stage to equilibrate the system at the target temperature 300 K, and finally, and 10 ps Production stage using a leap-frog Verlet integration algorithm, respectively.

### 3.6. General Procedure for the Synthesis of **2**

A mixture of 2-bromo-5-methoxyaniline (10 mmol, 2.02 g), malonic acid (15 mmol) and POCl_3_ (25 mL) was refluxed for 16 h. The reaction mixture was slowly poured into water and extracted with DCM. The organic layers was dried over Na_2_SO_4_, filtered, and concentrated under reduced pressure. The crude material was purified on silica gel, eluted with DCM, to afford the final product as a light yellow solid in 90% yield [8].^1^H-NMR (400 MHz, CDCl_3_): δ 7.96 (t, *J* = 8 Hz, 1H), 7.47 (s, 1H), 6.82 (t, *J* = 8 Hz, 1H), 3.96 (s, 3H). ^13^C-NMR (100 MHz, CDCl_3_): δ 156.2, 151.4, 144.2, 143.5, 138.0, 118.8, 122.0, 114.6, 105.0, 56.2. ESI-MS (*m/z*, %): 303.8989 [M−H]^−^. Mp: 204–206 °C.

### 3.7. General Procedure for the Synthesis of **3**

To 8-bromo-2,4-dichloro-5-methoxyquinoline (**2**, 10 mmol, 3.06 g) in 20 mL of THF, *n*-BuLi (11 mmol, 2.5 M, 4.5 mL) was added dropwise at −78 °C. The resulting mixture was stirred at −78 °C for 1 h, and CH_3_OH (2 mL) was added dropwise at −78 °C. The mixture was stirred at 0 °C for 1 h, and 20 mL of water and 20 mL of dichloromethane were added. The two layers were separated, and the aqueous layer was extracted with dichloromethane (3 × 30 mL). The combined organic extracts were washed with brine, dried over anhydrous sodium sulfate, filtered, and concentrated to afford the final product in 92% yield [9].^1^H-NMR (400 MHz, CDCl_3_): δ 7.64–7.47 (m, 2H), 7.34 (s, 1H), 6.88 (dd, *J* = 7.3, 1.3 Hz, 1H), 3.91 (s, 3H). ^13^C-NMR (100 MHz, CDCl_3_): δ 156.7, 150.4, 149.2, 143.6, 135.3, 121.6, 120.1, 118.2, 107.7, 56.2. ESI-MS (*m/z*, %): 225.9875 [M−H]^−^. Mp: 198–200 °C.

### 3.8. Synthesis of **4a**–**p**

The synthesis of compounds **4a**–**p** was performed according to the literature method [10].

### 3.9. General Procedure for the Synthesis of **5a**–**v**

To a solution of **4a**–**p** (0.2 mmol) in isopropanol (3 mL) was added various primary amines or secondary amines (0.4 mmol) and TFA (0.6 mmol, 68 mg). The resulting solution was stirred in a sealed vessel inside a microwave apparatus at 160 °C for 20 min. After cooling, TLC indicated the completion of the reaction. After removal of the solvent by rotary evaporation, the residue was redissolved in DCM, and washed with saturated NaHCO_3_ solution. The organic layer was dried, concentrated and purified by column chromatography (1% DCM-methanol) to give the desired compound [11].

*N-(1-Benzylpiperidin-4-yl)-5-methoxy-2-(4-methylpiperazin-1-yl)quinolin-4-amine* (**5a**): Yellow solid, Mp: 268–269 °C. 80% yield from **4a**. ^1^H-NMR (400 MHz, DMSO-*d_6_*): δ 7.79 (d, *J* = 7.6 Hz, 1H), 7.49 (t, *J* = 8.2 Hz, 1H), 7.38–7.29 (m, 4H), 7.21 (t, *J* = 6.9 Hz, 2H), 6.88 (d, *J* = 7.9 Hz, 1H), 6.39 (s, 1H), 3.94 (s, 3H), 3.79 (s, 3H), 3.32 (s, 4H), 3.07 (dd, *J* = 12.5Hz, 8.0 Hz, 6H), 2.79(s, 3H), 2.52 (s, 3H), 2.08 (s, 2H), 1.73 (d, *J* = 12.5 Hz, 1H). ^13^C-NMR (100 MHz, DMSO-*d_6_*): δ 174.2, 150.5, 149.8, 147.6, 138.6, 131.5, 128.8 (2C), 128.4 (2C), 127.2, 120.2, 105.7, 102.0, 94.6, 64.7, 57.2 (2C), 56.8, 56.2, 51.9 (2C), 47.6 (2C), 46.6, 30.3 (2C). ESI-MS: *m/z* 446.2875 [M+H]^+^.

*N-(1-Benzylpiperidin-4-yl)-5-methoxy-2-morpholinoquinolin-4-amine* (**5b**): Yellow solid, Mp: 248–249 °C. 82% yield from **4a**. ^1^H-NMR (400 MHz, DMSO-*d_6_*): δ 7.78 (d, *J* = 7.6 Hz, 1H), 7.52 (t, *J* = 8.2 Hz, 1H), 7.34–7.29 (m, 4H), 7.25 (t, *J* = 6.9 Hz, 2H), 6.91 (d, *J* = 7.9 Hz, 1H), 6.39 (s, 1H), 3.95 (s, 3H), 3.71(d, *J* = 4.1 Hz, 4H), 3.61(s, 4H), 3.06–2.88 (m, 2H), 2.01 (s, 2H), 1.64 (s, 2H), 1.20 (q, *J* = 8.0 Hz, 5H). ^13^C-NMR (100 MHz, DMSO-*d_6_*): δ 174.2, 150.5, 149.8, 147.6, 138.6, 131.5, 128.8 (2C), 128.4 (2C), 127.2, 120.2, 105.7, 102.0, 94.6, 66.3 (2C), 64.7, 56.8, 56.2, 51.9 (2C), 48.7 (2C), 30.3 (2C). ESI-MS: *m/z* 433.2559 [M+H]^+^.

*N-(1-Benzylpiperidin-4-yl)-5-methoxy-2-(piperidin-1-yl)quinolin-4-amine* (**5c**): Yellow solid, Mp: 244–246 °C. 85% yield from **4a**. ^1^H-NMR (400 MHz, DMSO-*d_6_*): δ 7.79 (d, *J* = 7.6 Hz, 1H), 7.45 (t, *J* = 8.2 Hz, 1H), 7.39–7.30 (m, 4H), 7.25 (t, *J* = 6.9 Hz, 2H), 6.88 (d, *J* = 7.9 Hz, 1H), 6.39 (s, 1H), 3.94 (s, 3H), 3.79–3.43 (m, 6H), 2.70 (s, 2H), 2.33 (s, 2H), 1.98 (s, 2H), 1.68 (s, 7H), 1.25(d, 2H). ^13^C-NMR (100 MHz, DMSO-*d_6_*): δ 174.2, 150.5, 149.8, 147.6, 138.6, 131.5, 128.8 (2C), 128.4 (2C), 127.2, 120.2, 105.7, 102.0, 94.6, 64.7, 56.8, 56.2, 51.9 (2C), 47.2 (2C), 30.3 (2C), 25.5 (2C), 24.5. ESI-MS: *m/z* 431.2766 [M+H]^+^.

*N4-(1-Benzylpiperidin-4-yl)-5-methoxy-N2-(pyrimidin-2-yl)quinoline-2,4-diamine* (**5d**): Yellow solid, Mp: 224–226 °C. 84% yield from **4a**. ^1^H-NMR (400 MHz, DMSO-*d_6_*): δ 7.58–7.29 (m, 10H), 6.86 (d, *J* = 8.0 Hz, 1H), 6.70 (d, *J* = 8.0 Hz, 1H), 5.29 (s, 1H), 4.44 (br s, 1H), 3.90 (s, 3H), 3.61 (s, 2H), 3.12–3.05 (m, 3H), 2.26 (d, *J* = 8.0 Hz, 2H), 2.09 (s, 2H), 1.73 (d, *J* = 8.0 Hz, 2H). ^13^C-NMR (100 MHz, DMSO-*d_6_*): δ 169.3, 157.9 (2C), 155.5, 148.4, 148.0, 146.0, 138.6, 129.9, 128.8 (2C), 128.4 (2C), 127.2, 117.8, 115.3, 104.6, 100.6, 94.5, 64.7, 56.8, 56.2, 51.9 (2C), 30.3 (2C). ESI-MS: *m/z* 441.2358 [M+H]^+^.

*N4-(1-Benzylpiperidin-4-yl)-5-methoxy-N2-(4-nitrophenyl)quinoline-2,4-diamine* (**5e**): Yellow solid, Mp: 226–228 °C. 88% yield from **4a**. ^1^H-NMR (400 MHz, DMSO-*d_6_*): δ 8.36 (d, *J* = 8.1 Hz, 2H), 8.19 (d, *J* = 8.6 Hz, 2H), 7.94 (d, *J* = 8.0 Hz, 1H), 7.81 (d, *J* = 8.0 Hz, 1H), 7.64 (d, *J* = 4.2 Hz, 1H), 7.35 (s, 2H), 6.73 (d, *J* = 8.8 Hz, 2H), 6.60 (d, *J* = 8.0 Hz, 1H), 6.13 (s, 1H), 3.96 (s, 3H), 3.54 (s, 2H), 2.25 (br s, 4H), 2.05 (br s, 4H). ^13^C-NMR (100 MHz, DMSO-*d_6_*): δ 155.5, 148.4, 148.0, 147.0, 146.0, 138.6, 137.9, 129.9, 128.8 (2C), 128.4 (2C), 127.2, 124.7 (2C), 119.2 (2C), 117.8, 104.6, 100.6, 94.5, 64.7, 56.8, 56.2, 51.9 (2C), 30.3 (2C). ESI-MS: *m/z* 484.2304 [M+H]^+^.

*N4-(1-Benzylpiperidin-4-yl)-N2-(3,5-bis(trifluoromethyl)phenyl)-5-methoxyquinoline-2,4-diamine* (**5f**): Yellow solid, Mp: 221–223 °C. 88% yield from **4a**. ^1^H-NMR (400 MHz, DMSO-*d_6_*): δ 10.79 (s, 2H), 7.54–7.29 (m, 6H), 7.40–7.20 (m, 2H), 6.86 (d, *J* = 8.1 Hz, 1H), 6.71 (d, *J* = 8.0 Hz, 1H), 5.34 (s, 1H) , 4.33 (s, 1H), 3.91 (s, 3H), 3.59 (s, 2H), 3.21–2.94 (m, 3H), 2.27 (d, *J* = 12.2 Hz, 2H), 2.08 (s, 2H), 1.68 (d, *J* = 12.2 Hz, 2H). ^13^C-NMR (100 MHz, DMSO-*d_6_*): δ 155.5, 148.4, 148.0, 146.0, 140.7, 138.6, 132.1 (2C), 129.9, 128.8 (2C), 128.4 (2C), 127.2, 124.2 (2C), 119.3 (2C), 117.8, 113.1, 104.6, 100.6, 94.5, 64.7, 56.8, 56.2, 51.9 (2C), 30.3 (2C). ESI-MS: *m/z* 575.2201 [M+H]^+^.

*N-(1-Benzylpiperidin-4-yl)-5-methoxy-2-(4-methyl-1,4-diazepan-1-yl)quinolin-4-amine* (**5g**): Yellow solid, Mp: 235–237 °C. 95% yield from **4a**. ^1^H-NMR (400 MHz, DMSO-*d_6_*): δ 7.34–7.04 (m, 9H), 6.42 (d, *J* = 7.4 Hz, 1H), 5.51 (s, 1H), 3.89 (s, 2H), 3.83 (s, 3H), 3.76–3.60 (m, 3H), 3.43 (br s, 1H), 2.70 (s, 4H), 2.53 (t, *J* = 4.0 Hz, 2H), 2.24 (t, *J* = 4.0 Hz , 2H), 2.09–1.89 (m, 5H), 1.82–1.46 (m, 4H). ^13^C-NMR (100 MHz, DMSO-*d_6_*): δ 174.2, 150.5, 149.8, 147.6, 138.6, 131.5, 128.8 (2C), 128.4 (2C), 127.2, 120.2, 105.7, 102.0, 94.6, 64.7, 61.8, 59.0, 57.6, 56.8, 56.2, 51.9 (3C), 46.9, 30.3 (2C), 26.4. ESI-MS: *m/z* 460.3032 [M+H]^+^.

*N-Isopentyl-5-methoxy-2-(4-methyl-1,4-diazepan-1-yl)quinolin-4-amine* (**5h**): Yellow solid, Mp: 215–217 °C. 80% yield from **4b**. ^1^H-NMR (400 MHz, DMSO-*d_6_*): δ 7.51 (d, *J* = 8.2 Hz, 2H), 7.27 (s, 1H), 6.74 (dd, *J* = 8.1, 2.2 Hz, 1H), 5.51 (s, 1H), 4.00 (s, 2H), 3.90 (s, 3H), 3.84 (t, *J* = 4.0 Hz, 2H), 3.74 (s, 1H), 3.29 (q, *J* = 8.0 Hz, 2H), 2.82 (t, *J* = 4.1 Hz, 2H), 2.62 (t, *J* = 4.0 Hz, 2H), 2.41(s, 3H), 2.10 (t, *J* = 8.1 Hz, 2H), 1.80–1.63 (m, 1H), 1.66 (q, *J* = 8.2 Hz, 2H), 1.22 (s, 3H), 1.00 (d, *J* = 8.1 Hz, 2H). ^13^C-NMR (100 MHz, DMSO-*d_6_*): δ 174.2 150.5, 149.8, 147.6, 131.5, 120.2, 105.7, 102.0, 94.6, 61.8, 59.0, 57.6, 56.2, 51.9, 46.9, 42.6, 26.4, 25.5, 22.9 (2C). ESI-MS: *m/z* 357.2610 [M+H]^+^.

*N-(1-Isopropylpiperidin-4-yl)-5-methoxy-2-(4-methyl-1,4-diazepan-1-yl)quinolin-4-amine* (**5i**): Yellow solid, Mp: 213–215 °C. 87% yield from **4c**. ^1^H-NMR (400 MHz, DMSO-*d_6_*): δ 7.37 (d, *J* = 4.0 Hz, 1H), 7.21 (t, *J* = 8.1 Hz, 1H), 6.86 (d, *J* = 8.3 Hz, 1H), 6.33 (d, *J* = 7.9 Hz, 1H), 5.63 (s, 1H), 3.91 (s, 3H), 3.79 (s, 1H), 3.64 (t, *J* = 7.9 Hz, 2H), 3.51–3.22 (m, 1H), 2.61 (s, 4H), 2.32–2.07 (m, 4H), 2.07–1.76 (m, 3H), 1.55 (d, *J* = 8.0 Hz, 2H), 1.35–1.13 (m, 4H), 1.07–0.89 (m, 6H), 0.84 (d, *J* = 7.4 Hz, 2H). ^13^C-NMR (100 MHz, DMSO-*d_6_*): δ 174.2, 150.5, 149.8, 147.6, 131.5, 120.2, 105.7, 102.0, 94.6, 61.8, 59.0, 58.3, 57.6, 56.8, 56.2, 51.9, 49.5 (2C), 46.9, 30.6 (2C), 26.4, 18.1. ESI-MS: *m/z* 412.3032 [M+H]^+^.

*5-Methoxy-2-(4-methyl-1,4-diazepan-1-yl)-N-(2-(4-methylpiperazin-1-yl)ethyl)quinolin-4-amine* (**5j**): Yellow solid, Mp: 210–212 °C. 86% yield from **4d**. ^1^H-NMR (400 MHz, DMSO-*d_6_*): δ 7.39 (d, *J* = 4.2 Hz, 1H), 7.23 (t, *J* = 7.9 Hz, 1H), 6.96 (d, *J* = 8.0 Hz, 1H), 6.53 (d, *J* = 8.1 Hz, 1H), 3.86 (s, 3H), 3.79 (s, 1H), 3.64 (t, *J* = 8.1 Hz, 2H), 3.51 (s, 3H), 3.45–3.21 (m, 2H), 2.61 (s, 4H), 2.53–2.07 (m, 6H), 2.03–1.65 (m, 3H), 1.47 (d, *J* = 7.8 Hz, 2H), 1.35–0.98 (m, 4H), 0.78 (d, *J* = 7.4 Hz, 2H). ^13^C-NMR (100 MHz, DMSO-*d_6_*): δ 174.2, 150.5, 149.8, 147.6, 131.5, 120.2, 105.7, 102.0, 94.6, 61.8, 59.0, 57.6 (5C), 56.2, 55.5, 51.9, 47.6, 47.6, 46.9, 46.6, 26.4. ESI-MS: *m/z* 413.2984 [M+H]^+^.

*5-Methoxy-2-(4-methyl-1,4-diazepan-1-yl)-N-(1-methylpiperidin-4-yl)quinolin-4-amine* (**5k**): Yellow solid, Mp: 212–220 °C. 86% yield from **4e**. ^1^H-NMR (400 MHz, DMSO-*d_6_*): δ 7.40 (d, *J* = 4.0 Hz, 1H), 7.22 (t, *J* = 8.0 Hz, 1H), 6.96 (d, *J* = 8.1 Hz, 1H), 6.54 (d, *J* = 8.3 Hz, 1H), 5.69 (s, 1H), 3.91 (s, 3H), 3.79 (s, 2H), 3.64 (t, *J* = 8.5 Hz, 2H), 3.54 (s, 1H), 3.44 (q, *J* = 7.0 Hz, 1H), 2.71–2.55 (m, 4H), 2.29 (t, *J* = 3.4 Hz, 5H), 2.25–2.18 (m, 3H), 1.98 (d, *J* = 10.2 Hz, 2H), 1.94–1.85 (m, 2H), 1.56 (dd, *J* = 18.3, 9.0 Hz, 2H), 1.06 (t, *J* = 7.0 Hz, 1H). ^13^C-NMR (100 MHz, DMSO-*d_6_*): δ 157.3, 157.2, 151.2,150.6, 128.5, 119.6, 105.6, 100.7, 83.1, 57.8, 56.5, 56.0(2C), 53.1, 45.9(4C), 45.3, 30.5, 27.0, 18. ESI-MS: *m/z* 384.2719 [M+H]^+^.

*5-Methoxy-2-(4-methyl-1,4-diazepan-1-yl)-N-(tetrahydro-2H-pyran-4-yl)quinolin-4-amine* (**5l**): Yellow solid, Mp: 213–215 °C. 86% yield from **4f**. ^1^H-NMR (400 MHz, DMSO-*d_6_*): δ 8.41 (s, 1H), 7.81 (s, 1H), 7.66 (s, 1H), 7.04 (s, 1H), 5.77 (s, 1H), 4.32 (s, 1H), 4.03 (s, 4H), 3.94–3.65 (m, 3H), 3.56 (t, *J* = 10.4 Hz, 3H), 3.37 (d, *J* = 7.4 Hz, 1H), 3.37 (d, *J* = 7.4 Hz, 1H), 3.07 (s, 1H), 2.80 (s, 2H), 2.22 (s, 1H), 1.99 (d, *J* = 12.3 Hz, 2H), 1.64 (d, *J* = 9.1 Hz, 2H), 1.20 (dd, *J* = 16.0, 8.6 Hz, 3H), 0.85 (s, 1H). ^13^C-NMR (100 MHz, DMSO-*d_6_*): δ 174.2, 150.5, 149.8, 147.6, 131.5, 120.2, 105.7, 102.0, 94.6, 65.5 (2C), 61.8, 59.0, 57.6, 56.2, 55.5, 51.9, 46.9, 36.8 (2C), 26.4. ESI-MS: *m/z* 371.2402 [M+H]^+^.

*5-Methoxy-N-(4-methoxyphenyl)-2-(4-methyl-1,4-diazepan-1-yl)quinolin-4-amine* (**5m**): Yellow solid, Mp: 268–270 °C. 81% yield from **4g**. ^1^H-NMR (400 MHz, DMSO-*d_6_*): δ 8.85 (s, 1H), 7.70 (s, 1H), 7.42 (t, *J* = 7.5 Hz, 1H), 7.18 (d, *J* = 8.2 Hz, 2H), 6.91 (d, *J* = 8.5 Hz, 2H), 6.75 (d, *J* = 7.7 Hz, 1H), 6.33 (s, 1H), 3.88 (s, 3H), 3.74 (s, 3H), 3.49 (br s, 4H), 3.16 (t, *J* = 8.5 Hz, 2H), 2.85 (s, 3H), 2.59 (t, *J* = 8.5 Hz, 2H), 1.23–1.08 (m, 2H). ^13^C-NMR (100 MHz, DMSO-*d_6_*): δ 155.8, 153.3, 153.1, 150.1, 149.5, 133.2, 130.6, 121.7 (2C), 118.5, 115.1 (2C), 110.9, 101.6, 99.8, 61.8, 59.0, 58.0, 56.2, 55.8, 52.3, 46.9, 26.4. ESI-MS: *m/z* 393.2246 [M+H]^+^.

*N-(4-Isopropylphenyl)-5-methoxy-2-(4-methyl-1,4-diazepan-1-yl)quinolin-4-amine* (**5n**): Yellow solid, Mp: 278–280 °C. 81% yield from **4h**. ^1^H-NMR (400 MHz, DMSO-*d_6_*): δ 8.82 (s, 1H),7.62 (d, *J* = 9.0 Hz, 2H), 7.35 (t, *J* = 8.1 Hz, 1H), 7.11 (d, *J* = 8.2 Hz, 1H), 6.73 (d, *J* = 9.0 Hz, 2H), 6.67 (d, *J* = 7.9 Hz, 1H), 6.32 (s, 1H), 3.85 (s, 3H), 3.49 (br s, 4H), 3.16 (t, *J* = 8.5 Hz, 2H), 2.88 (s, 1H), 2.85 (s, 3H), 2.59 (t, *J* = 8.5 Hz, 2H), 1.20 (d, *J* = 8.5 Hz, 6H), 1.08–0.97 (m, 2H). ^13^C-NMR (100 MHz, DMSO-*d_6_*): δ 155.8, 153.1, 150.1, 149.5, 138.4, 138.1, 130.6, 126.9 (2C), 120.1 (2C), 118.5, 110.9, 101.6, 99.8, 61.8, 59.0, 58.0, 56.2, 52.3, 46.9, 33.2, 26.4, 23.3 (2C). ESI-MS: *m/z* 405.2610 [M+H]^+^.

*5-Methoxy-N-(2-methoxyphenyl)-2-(4-methyl-1,4-diazepan-1-yl)quinolin-4-amine* (**5o**): Yellow solid, Mp: 265–267 °C. 81% yield from **4i**. ^1^H-NMR (400 MHz, DMSO-*d_6_*): δ 8.89 (s, 1H), 8.67 (s, 1H), 8.23 (s, 1H), 7.38 (t, *J* = 7.5 Hz, 1H), 7.18 (d, *J* = 7.8 Hz, 1H), 7.01 (s, 1H), 6.93 (s, 2H), 6.73 (s, 1H), 3.87 (s, 6H), 3.49 (br s, 4H), 3.16 (t, *J* = 8.5 Hz, 2H), 2.85 (s, 3H), 2.59 (t, *J* = 8.5 Hz, 2H), 1.23–1.08 (m, 2H). ^13^C-NMR (100 MHz, DMSO-*d_6_*): δ 155.8, 153.1, 150.1, 149.5, 147.4, 132.6, 130.6, 122.6, 121.8, 118.5, 113.4, 112.0, 110.9, 101.6, 99.8, 61.8, 59.0, 58.0, 56.2, 55.8, 52.3, 46.9, 26.4. ESI-MS: *m/z* 393.2246 [M+H]^+^.

*N1-(5-Methoxy-2-(4-methyl-1,4-diazepan-1-yl)quinolin-4-yl)-N4,N4-dimethylbenzene-1,4-diamine* (**5p**): Yellow solid, Mp: 268–270 °C. 81% yield from **4l**. ^1^H-NMR (400 MHz, DMSO-*d_6_*): δ 8.82 (s, 1H),7.62 (d, *J* = 9.0 Hz, 2H), 7.35 (t, *J* = 8.1 Hz, 1H), 7.11 (d, *J* = 8.2 Hz, 1H), 6.73 (d, *J* = 9.0 Hz, 2H), 6.67 (d, *J* = 7.9 Hz, 1H), 6.32 (s, 1H), 3.85 (s, 3H), 3.49 (br s, 4H), 3.16 (t, *J* = 8.5 Hz, 2H), 3.02 (s, 6H), 2.85 (s, 3H), 2.59 (t, *J* = 8.5 Hz, 2H), 1.08–0.97 (m, 2H). ^13^C-NMR (100 MHz, DMSO-*d_6_*): δ 155.8, 153.1, 150.1, 149.5, 138.4, 138.1, 130.6, 126.9 (2C), 120.1 (2C), 118.5, 110.9, 101.6, 99.8, 61.8, 59.0, 58.0, 56.2, 52.3, 46.9, 26.4, 23.3 (2C). ESI-MS: *m/z* 406.2562 [M+H]^+^.

*5-Methoxy-N-(4-methoxy-2-methylphenyl)-2-(4-methyl-1,4-diazepan-1-yl)quinolin-4-amine* (**5q**): Yellow solid, Mp: 258–260 °C. 88% yield from **4j**. ^1^H-NMR (400 MHz, DMSO-*d_6_*): δ 8.85 (s, 1H), 7.70 (s, 2H), 7.42 (t, *J* = 7.5 Hz, 1H), 7.18 (d, *J* = 8.2 Hz, 1H), 6.91 (d, *J* = 8.5 Hz, 1H), 6.75 (d, *J* = 7.7 Hz, 1H), 6.33 (s, 1H), 3.88 (s, 3H), 3.74 (s, 3H), 3.49 (br s, 4H), 3.16 (t, *J* = 8.5 Hz, 2H), 2.85 (s, 3H), 2.59 (t, *J* = 8.5 Hz, 2H), 2.12 (s, 3H), 1.23–1.08 (m, 2H). ^13^C-NMR (100 MHz, DMSO-*d_6_*): δ 155.8, 153.1, 151.1, 150.1, 149.5, 134.3, 130.6, 130.0, 121.4, 118.5, 117.2, 112.1, 110.9, 101.6, 99.8, 66.3 (2C), 56.2, 55.8, 53.7 (2C), 17.9. ESI-MS: *m/z* 407.2402 [M+H]^+^.

*5-Methoxy-2-(4-methyl-1,4-diazepan-1-yl)-N-(p-tolyl)quinolin-4-amine* (**5r**): Yellow solid, Mp: 246–248 °C. 88% yield from **4m**. ^1^H-NMR (400 MHz, DMSO-*d_6_*): δ 7.23 (d, *J* = 9.0 Hz, 4H), 7.35 (t, *J* = 8.1 Hz, 1H), 7.11 (d, *J* = 8.2 Hz, 1H), 6.73 (d, *J* = 9.0 Hz, 1H), 6.67 (d, *J* = 7.9 Hz, 1H), 6.32 (s, 1H), 3.85 (s, 3H), 3.51 (s, 2H), 3.45 (br s, 2H), 3.03–2.88 (m, 2H), 2.84 (s, 6H), 2.58 (s, 2H), 1.23–1.08 (m, 2H). ^13^C-NMR (100 MHz, DMSO-*d_6_*): δ 155.8, 153.3, 153.1, 150.1, 149.5, 133.2, 131.2, 129.8 (2C), 118.5, 116.3 (2C), 110.9, 101.6, 99.8, 61.8, 59.0, 58.0, 56.2, 52.3, 46.9, 26.4, 21.3. ESI-MS: *m/z* 377.2297 [M+H]^+^.

*5-Methoxy-N-(3-methoxyphenyl)-2-(4-methyl-1,4-diazepan-1-yl)quinolin-4-amine* (**5s**): Yellow solid, Mp: 244–246 °C. 86% yield from **4k**. ^1^H-NMR (400 MHz, DMSO-*d_6_*): δ7.73–7.45 (m, 2H), 7.25 (dd, *J* = 14.5 HZ, 8.0 HZ, 3H), 6.85 (d, *J* =7.5 HZ, 1H), 6.62 (s, 1H), 6.58 (s, 1H), 6.47 (s, 1H), 3.90 (s, 3H), 3.51 (s, 2H), 3.45 (br s, 2H), 3.03–2.88 (m, 2H), 2.84 (s, 6H), 2.58 (s, 2H), 1.23–1.08 (m, 2H). ^13^C-NMR (100 MHz, DMSO-*d_6_*): δ 155.8, 153.1, 150.1, 149.5, 147.4, 132.6, 130.6, 122.6, 121.8, 118.5, 113.4, 112.0, 110.9, 101.6, 99.8, 61.8, 59.0, 58.0, 56.2, 55.8, 52.3, 46.9, 26.4. ESI-MS: *m/z* 393.2246 [M+H]^+^.

*5-Methoxy-2-(4-methyl-1,4-diazepan-1-yl)-N-(pyrimidin-5-yl)quinolin-4-amine* (**5t**): Yellow solid, Mp: 254–256 °C. 88% yield from **4n**. ^1^H-NMR (400 MHz, DMSO-*d_6_*): δ 8.90 (s, 1H), 8.47 (d, *J* = 8.5 Hz, 2H), 7.19–7.17 (m, 2H), 6.91 (s, 1H), 6.87 (t, *J* = 8.5 Hz, 1H), 6.19 (d, *J* = 8.5 Hz, 1H), 3.90 (s, 3H), 3.49 (br s, 4H), 3.16 (t, *J* = 8.5 Hz, 2H), 2.85 (s, 3H), 2.59 (t, *J* = 8.5 Hz, 2H), 1.23–1.08 (m, 2H). ^13^C-NMR (100 MHz, DMSO-*d_6_*): δ 174.2, 150.5, 147.6, 147.4, 144.8, 143.7 (2C), 142.9, 131.5, 120.2, 105.7, 102.0, 94.6, 61.8, 59.0, 57.6, 56.2, 51.9, 46.9, 26.4. ESI-MS: *m/z* 365.2045 [M+H]^+^.

*5-Methoxy-2-(4-methyl-1,4-diazepan-1-yl)-N-(4-nitrophenyl)quinolin-4-amine* (**5u**): Yellow solid, Mp: 283–285 °C. 88% yield from **4o**. ^1^H-NMR (400 MHz, DMSO-*d_6_*): δ 8.85 (s, 1H), 8.03 (d, *J* = 8.5 Hz, 2H), 7.70 (s, 1H), 7.42 (t, *J* = 7.5 Hz, 1H), 7.18 (d, *J* = 8.2 Hz, 2H), 6.75 (d, *J* = 7.7 Hz, 1H), 6.33 (s, 1H), 3.90 (s, 3H), 3.49 (br s, 4H), 3.16 (t, *J* = 8.5 Hz, 2H), 2.85 (s, 3H), 2.59 (t, *J* = 8.5 Hz, 2H), 1.23–1.08 (m, 2H). ^13^C-NMR (100 MHz, DMSO-*d_6_*): δ 174.2, 152.0, 150.5, 147.6, 144.8, 137.9, 131.5, 124.7 (2C), 120.2, 119.2 (2C), 105.7, 102.0, 94.6, 61.8, 59.0, 57.6, 56.2, 51.9, 46.9, 26.4. ESI-MS: *m/z* 408.1991 [M+H]^+^.

*N-(3,5-Bis(trifluoromethyl)phenyl)-5-methoxy-2-(4-methyl-1,4-diazepan-1-yl)quinolin-4-amine* (**5v**): Yellow solid, Mp: 297–299 °C. 78% yield from **4p**. ^1^H-NMR (400 MHz, DMSO-*d_6_*): δ 8.85 (s, 1H), 8.03 (d, *J* = 8.5 Hz, 2H), 7.56 (s, 2H), 7.49 (s, 1H), 7.42 (t, *J* = 7.5 Hz, 1H), 6.33 (s, 1H), 3.90 (s, 3H), 3.49 (br s, 4H), 3.16 (t, *J* = 8.5 Hz, 2H), 2.85 (s, 3H), 2.59 (t, *J* = 8.5 Hz, 2H), 1.23–1.08 (m, 2H). ^13^C-NMR (100 MHz, DMSO-*d_6_*): δ 174.2, 150.5, 147.6, 144.8, 143.0, 132.1 (2C), 131.5, 124.4 (2C), 120.2, 119.3 (2C), 113.1, 105.7, 102.0, 94.6, 61.8, 59.0, 57.6, 56.2, 51.9, 46.9, 26.4. ESI-MS: *m/z* 499.1888 [M+H]^+^.

### 3.10. Synthesis of **7a**–**i**

Compounds **7a**‒**i** were synthesized according to the literature method [8].

### 3.11. Synthesis of **8a**–**i**

Compounds **8a**‒**i** were synthesized according to the literature method [10].

### 3.12. General Procedure for the Synthesis of **9a**–**i**

The general procedure for the synthesis of **9a**–**i** was the same as that described for **5a**–**v**.

*6-Methoxy-2-(4-methyl-1,4-diazepan-1-yl)-N-(1-methylpiperidin-4-yl)quinolin-4-amine* (**9a**): Yellow solid, Mp: 266–268 °C. 75% yield from **8a**. ^1^H-NMR (400 MHz, DMSO-*d_6_*): δ 7.55 (d, *J* = 8 Hz, 1H), 7.26 (s, 1H), 6.97 (d, *J* = 8 Hz, 1H), 6.36 (s, 1H), 5.65 (s, 1H), 3.81 (s, 3H), 3.79 (s, 1H), 3.66 (t, *J* = 8 Hz, 2H), 3.48 (s, 3H), 2.61 (s, 4H), 2.25–2.05 (m, 4H), 2.05–1.76 (m, 3H), 1.58 (d, *J* = 8 Hz, 2H), 1.35–0.98 (m, 4H), 0.84 (d, *J* = 7.4 Hz, 2H). ^13^C-NMR (100 MHz, DMSO-*d_6_*): δ 171.7, 154.7, 152.5, 145.4, 129.6 122.7, 116.7, 101.6, 96.0, 61.8, 59.0, 57.6, 56.8, 56.2, 54.5 (2C), 51.9, 46.9, 30.3 (2C), 26.4. ESI-MS: *m/z* 384.2719 [M+H]^+^.

*7-Methoxy-2-(4-methyl-1,4-diazepan-1-yl)-N-(1-methylpiperidin-4-yl)quinolin-4-amine* (**9b**): Yellow solid, Mp: 261–263 °C. 65% yield from **8b**. ^1^H-NMR (400 MHz, DMSO-*d_6_*): δ 7.42 (d, *J* = 8 Hz, 1H), 7.09 (s, 1H), 6.85 (d, *J* = 8 Hz, 1H), 6.40 (s, 1H), 5.65 (s, 1H), 3.87 (s, 3H), 3.79 (s, 1H), 3.62 (t, *J* = 8 Hz, 2H), 3.48 (s, 3H), 2.65 (s, 4H), 2.15–2.05 (m, 4H), 2.05–1.66 (m, 3H), 1.45 (d, *J* = 8 Hz, 2H), 1.35–0.88 (m, 4H), 0.84 (d, *J* = 7.4 Hz, 2H). ^13^C-NMR (100 MHz, DMSO-*d_6_*): δ 174.4, 155.6, 150.7, 147.8, 122.5, 112.9, 109.2, 106.4, 93.3, 61.8, 59.0, 57.6, 56.8, 56.2, 54.5 (2C), 51.9, 46.9, 30.3 (2C), 26.4. ESI-MS: *m/z* 384.2719 [M+H]^+^.

*8-Methoxy-2-(4-methyl-1,4-diazepan-1-yl)-N-(1-methylpiperidin-4-yl)quinolin-4-amine* (**9c**): Yellow solid, Mp: 251–253 °C. 69% yield from **8c**. ^1^H-NMR (400 MHz, DMSO-*d_6_*): δ 8.01 (d, *J* = 8 Hz, 1H), 7.33 (s, 1H), 7.17 (d, *J* = 8 Hz, 1H), 6.38 (s, 1H), 5.65 (s, 1H), 4.07 (s, 3H), 3.79 (s, 1H), 3.68 (t, *J* = 8 Hz, 2H), 3.56 (s, 3H), 2.45 (s, 4H), 2.18–2.05 (m, 4H), 2.05–1.66 (m, 3H), 1.45 (d, *J* = 8 Hz, 2H), 1.35–0.88 (m, 4H), 0.84 (d, *J* = 7.4 Hz, 2H). ^13^C-NMR (100 MHz, DMSO-*d_6_*): δ 173.0, 155.8, 155.2, 141.6, 117.4, 116.5, 116.3, 107.5, 95.8, 61.8, 59.0, 57.6, 56.8, 56.2, 54.5 (2C), 51.9, 46.9, 30.3 (2C), 26.4. ESI-MS: *m/z* 384.2717 [M+H]^+^.

*5,6-Dimethoxy-2-(4-methyl-1,4-diazepan-1-yl)-N-(1-methylpiperidin-4-yl)quinolin-4-amine* (**9d**): Yellow solid, Mp: 272–274 °C. 73% yield from **8d**. ^1^H-NMR (400 MHz, DMSO-*d_6_*): δ 7.00 (d, *J* = 8 Hz, 1H), 6.81 (s, 1H), 6.23 (d, *J* = 8 Hz, 1H), 5.65 (s, 1H), 3.83 (s, 6H), 3.79 (s, 1H), 3.68 (t, *J* = 8 Hz, 2H), 3.56 (s, 3H), 2.45 (s, 4H), 2.19–2.09 (m, 4H), 2.09–1.66 (m, 3H), 1.43 (d, *J* = 8 Hz, 2H), 1.35–0.88 (m, 4H), 0.84 (d, *J* = 7.4 Hz, 2H). ^13^C-NMR (100 MHz, DMSO-*d_6_*): δ 172.0, 151.1, 148.6, 146.2, 133.8, 122.5, 121.7, 106.7, 95.1, 61.8, 59.0, 57.6, 56.8, 56.2, 55.8, 54.5 (2C), 51.9, 46.9, 30.3 (2C), 26.4. ESI-MS: *m/z* 414.2824 [M+H]^+^.

*5,7-Dimethoxy-2-(4-methyl-1,4-diazepan-1-yl)-N-(1-methylpiperidin-4-yl)quinolin-4-amine* (**9e**): Yellow solid, Mp: 272–274 °C. 73% yield from **8e**. ^1^H-NMR (400 MHz, DMSO-*d_6_*): δ 6.54 (s, 1H), 6.27 (s, 1H), 6.22 (s, 1H), 5.65 (s, 1H), 3.81 (s, 6H), 3.77 (s, 1H), 3.69 (t, *J* = 8 Hz, 2H), 3.57 (s, 3H), 2.45 (s, 4H), 2.19–2.09 (m, 4H), 2.09–1.66 (m, 3H), 1.43 (d, *J* = 8 Hz, 2H), 1.35–0.88 (m, 4H), 0.84 (d, *J* = 7.4 Hz, 2H). ^13^C-NMR (100 MHz, DMSO-*d_6_*): δ 174.4, 155.8, 154.7, 151.5, 149.5, 101.4, 98.5, 94.6, 92.4, 61.8, 59.0, 57.6, 56.8, 56.2, 55.8, 54.5 (2C), 51.9, 46.9, 30.3 (2C), 26.4. ESI-MS: *m/z* 414.2822 [M+H]^+^.

*5,8-Dimethoxy-2-(4-methyl-1,4-diazepan-1-yl)-N-(1-methylpiperidin-4-yl)quinolin-4-amine* (**9f**): Yellow solid, Mp: 276–278 °C. 69% yield from **8f**. ^1^H-NMR (400 MHz, DMSO-*d_6_*): δ 6.25 (s, 1H), 5.97 (d, *J* = 8 Hz, 1H), 5.88 (d, *J* = 8 Hz, 1H), 5.65 (s, 1H), 3.80 (s, 6H), 3.77 (s, 1H), 3.69 (t, *J* = 8 Hz, 2H), 3.57 (s, 3H), 2.45 (s, 4H), 2.19–2.09 (m, 4H), 2.09–1.66 (m, 3H), 1.43 (d, *J* = 8 Hz, 2H), 1.35–0.88 (m, 4H), 0.84 (d, *J* = 7.4 Hz, 2H). ^13^C-NMR (100 MHz, DMSO-*d_6_*): δ 173.3, 149.1, 147.9, 142.4, 139.7, 107.3, 106.5, 99.3, 94.9, 61.8, 59.0, 57.6, 56.8, 56.2, 55.8, 54.5 (2C), 51.9, 46.9, 30.3 (2C), 26.4. ESI-MS: *m/z* 414.2824 [M+H]^+^.

*6,7-Dimethoxy-2-(4-methyl-1,4-diazepan-1-yl)-N-(1-methylpiperidin-4-yl)quinolin-4-amine* (**9g**): Yellow solid, Mp: 253–255 °C. 69% yield from **8g**. ^1^H-NMR (400 MHz, DMSO-*d_6_*): δ 6.73 (s, 1H), 6.54 (s, 1H), 6.26 (s, 1H), 5.65 (s, 1H), 3.80 (s, 6H), 3.77 (s, 1H), 3.69 (t, *J* = 8 Hz, 2H), 3.57 (s, 3H), 2.45 (s, 4H), 2.19–2.09 (m, 4H), 2.09–1.66 (m, 3H), 1.43 (d, *J* = 8 Hz, 2H), 1.35–0.88 (m, 4H), 0.84 (d, *J* = 7.4 Hz, 2H). ^13^C-NMR (100 MHz, DMSO-*d_6_*): δ 172.2, 154.4, 151.2, 146.4, 145.6, 112.4, 105.8, 100.0, 93.8, 61.8, 59.0, 57.6, 56.8, 56.1 (2C), 54.5 (2C), 51.9, 46.9, 30.3 (2C), 26.4. ESI-MS: *m/z* 414.2820 [M+H]^+^.

*6,8-Dimethoxy-2-(4-methyl-1,4-diazepan-1-yl)-N-(1-methylpiperidin-4-yl)quinolin-4-amine* (**9h**): Yellow solid, Mp: 258–260 °C. 73% yield from **8h**. ^1^H-NMR (400 MHz, DMSO-*d_6_*): δ 6.71 (s, 1H), 6.33 (s, 1H), 6.24 (s, 1H), 5.65 (s, 1H), 3.93 (s, 6H), 3.77 (s, 1H), 3.69 (t, *J* = 8 Hz, 2H), 3.57 (s, 3H), 2.45 (s, 4H), 2.19–2.09 (m, 4H), 2.09–1.66 (m, 3H), 1.43 (d, *J* = 8 Hz, 2H), 1.35–0.88 (m, 4H), 0.84 (d, *J* = 7.4 Hz, 2H). ^13^C-NMR (100 MHz, DMSO-*d_6_*): δ 170.8, 156.1, 154.8, 154.0, 137.3, 117.5, 96.3, 93.7, 93.0, 61.8, 59.0, 57.6, 56.8, 56.1 (2C), 55.8 (2C), 51.9, 46.9, 30.3 (2C), 26.4. ESI-MS: *m/z* 414.2824 [M+H]^+^.

*7,8-Dimethoxy-2-(4-methyl-1,4-diazepan-1-yl)-N-(1-methylpiperidin-4-yl)quinolin-4-amine* (**9i**): Yellow solid, Mp: 258–260 °C. 73% yield from **8i**. ^1^H-NMR (400 MHz, DMSO-*d_6_*): δ 6.87 (d, *J* = 8 Hz, 1H), 6.70 (d, *J* = 8 Hz, 1H), 6.28 (s, 1H), 5.65 (s, 1H), 3.83 (s, 6H), 3.77 (s, 1H), 3.69 (t, *J* = 8 Hz, 2H), 3.57 (s, 3H), 2.45 (s, 4H), 2.19–2.09 (m, 4H), 2.09–1.66 (m, 3H), 1.43 (d, *J* = 8 Hz, 2H), 1.35–0.88 (m, 4H), 0.84 (d, *J* = 7.4 Hz, 2H). ^13^C-NMR (100 MHz, DMSO-*d_6_*): δ 173.5, 154.9, 142.6, 141.8 (2C), 114.6, 112.2, 108.4, 93.6, 93.0, 61.8, 60.9, 59.0, 57.6, 56.1, 55.8 (2C), 51.9, 46.9, 30.3 (2C), 26.4. ESI-MS: *m/z* 414.2824 [M+H]^+^.

## 4. Conclusions

In summary, a series of quinoline derivatives were synthesized, and SAR analysis led to the discovery of a number of compounds that showed good activities in both enzymatic and cellular assays. The most active compound, **5k**, exhibited an IC_50_ value of 1.2 μM against EZH2, decreased global H3K27me3 level in cells and also showed good selectivity for EZH2 against several selected protein methyltransferases. Compound **5k** could be a lead compound deserving further structural optimization due to the fact that it represents a new scaffold and possesses a low molecular weight.
